# Positive change in asthma control using therapeutic patient education in severe uncontrolled asthma: a one-year prospective study

**DOI:** 10.1186/s40733-021-00076-y

**Published:** 2021-07-21

**Authors:** Xiaoxian Zhang, Zhengdao Lai, Rihuang Qiu, E Guo, Jing Li, Qingling Zhang, Naijian Li

**Affiliations:** 1 Department of Allergy and Clinical Immunology, State Key Laboratory of Respiratory Disease, National Clinical Research Center for Respiratory Disease, Guangzhou Institute of Respiratory Health, the First Affiliated Hospital of Guangzhou Medical University, 151 Yan Jiang Rd, 510000 Guangzhou, P.R. China; 2 Department of Pulmonary and Critical Care Medicine, Dongguan People’s Hospital, 523000 Dongguan, P.R. China; 3 Department of Respiratory Medicine, The Affiliated Ganzhou Hospital of Nanchang University, 341000 Ganzhou, P.R. China; 4 Xiangyang Central Hospital, Hubei University of Arts and Science, 441000 Xiangyang, P.R. China

**Keywords:** Severe uncontrolled asthma, Therapeutic patient education, Asthma control

## Abstract

**Background:**

Severe asthma is difficult to control. Therapeutic patient education enables patients to better understand their disease and cope with treatment, but the effect of therapeutic patient education in severe uncontrolled asthma is unclear. We evaluated whether therapeutic patient education is effective in improving asthma control and decreasing the frequency of exacerbations in severe uncontrolled asthma.

**Methods:**

This was a prospective, observational, and self-controlled study that enrolled 40 subjects with severe uncontrolled asthma. Patients were seen at a clinic four times (on day 1 and after 3, 6, and 12 months). After baseline data collection, the subjects completed a therapeutic patient education program and were also followed-up via telephone after 1, 2, 4, 5, 7, 8, 9, 10, and 11 months to monitor asthma medication adherence and collect asthma-related information.

**Results:**

Within the 1-year study period, a total of 23 exacerbations were recorded in 14 patients, seven of whom required emergency treatment and two of whom were hospitalized. Twelve months after the standardized therapeutic patient education program, pulmonary function and fractional exhaled nitric oxide levels improved significantly in all 40 patients. Moreover, the scores from three standardized asthma questionnaires and indices suggested improved quality of life in these patients with severe uncontrolled asthma. Serum levels of biomarkers reflecting asthma immune responses did not change between baseline and the 1-year follow-up time point.

**Conclusions:**

Therapeutic patient education is effective in improving asthma control and decreasing exacerbations in patients with severe uncontrolled asthma.

**Supplementary Information:**

The online version contains supplementary material available at 10.1186/s40733-021-00076-y.

## Background

Asthma is a worldwide problem that currently affects an estimated 358 million individuals [1]. Over the past decade, the aims of asthma management have altered to focus on achieving and maintaining good asthma control and reducing future risks, such as decreases in lung function, asthma exacerbations, hospitalizations, adverse effects from treatment, and death [2]. Despite the widespread dissemination of asthma management guidelines, many patients have inadequately controlled disease and experience frequent asthma exacerbations [3, 4]. Patients who experience symptoms and severe asthma attacks even with high-dose inhaled corticosteroids plus another controller medication and/or systemic corticosteroids are defined as having severe uncontrolled asthma [3, 5]. Between 2.3 and 3.6 % of patients with persistent asthma have severe uncontrolled asthma [6], but this minority accounts for substantially greater asthma-related healthcare resource use and costs than the majority [7].

Treatment strategies for severe uncontrolled asthma have focused on the use of anti-inflammatory and bronchodilator drugs [3]. Despite multiple treatment options, therapy for severe uncontrolled asthma remains a challenge. Asthma control is affected by multiple factors, which also require prevention measures to decrease negative behaviors and aggravating factors [8, 9]. Previous studies provide evidence for the efficacy and efficiency of patient education and asthma self-management [10, 11]; therapeutic patient education, which helps patients acquire or maintain the skills they need to self-manage a chronic disease [12], has been well-studied in asthma control in children [13]. However, whether therapeutic patient education is effective in improving asthma control in severe uncontrolled asthma cases is unknown. The aim of this study was to investigate the effect of therapeutic patient education on asthma control, exacerbations, pulmonary function, quality of life, and serum biomarkers in patients with severe uncontrolled asthma.

## Methods

### Study design

We conducted a prospective single-center study in the Department of Allergy and Clinical Immunology, the First Affiliated Hospital of Guangzhou Medical University (Guangzhou, China). All patients provided written informed consent and the study was approved by the Chinese Clinical Trial Registry (ChiCTR-TRC-12,002,527). A total of 40 subjects with severe uncontrolled asthma were included in the study, to be seen at the clinic four times over 12 months. On visit 1 (day 1), the subjects were screened and baseline data were collected, including demographic characteristics, medical history, serum biomarker levels, asthma-specific assessment, asthma-related healthcare use, and an asthma assessment questionnaire. After baseline data collection, the subjects then completed therapeutic patient education. All subjects were required to attend three follow-up visits at 3 (visit 2), 6 (visit 3), and 12 (visit 4) months. All subjects were also contacted by telephone after 1, 2, 4, 5, 7, 8, 9, 10, and 11 months to monitor asthma medication adherence and collect asthma-related information. Table 1 details the study design.

**Table 1:** Schedule of assessments

Assessment/procedure	visit				
	1	2	3	4	Telephone visit
	Day 1	3 months after Day 1	6 months after Day 1	12 months after Day 1	1,2,4,5,7,8,9,10 and 11 months after Day 1
Medical history	✓				
TPE	✓	✓	✓	✓	
Spirometry	✓		✓	✓	
Exacerbation assessment	✓	✓	✓	✓	✓
PRO	✓	✓	✓	✓	
FeNO	✓		✓	✓	
Biomarker test	✓		✓	✓	

*TPE* therapeutic patient education; *PRO* patient report outcomes (Asthma Control Questionnaire 5, Standardized Asthma Quality of Life Questionnaire, and Asthma Symptom Utility Index); *FeNO* fractional exhaled nitric oxide

### Patients

Patients with severe uncontrolled asthma were defined as subjects whose asthma was inadequately controlled, as assessed by the five-item Asthma Control Questionnaire (ACQ-5) score of ≥ 1.5 with one or more uncontrolled asthma symptoms consistent with the Global Initiative for Asthma guidelines (nighttime awakening ≥ 1 time/week, symptoms > 2 days/week, short-acting beta-agonist use > 2 days/week, or interference with daily activities) despite daily use of inhaled corticosteroid corresponding to 500–2,000 µg/day of fluticasone propionate dry powder inhaler or the equivalent and a second eligible asthma controller medication [2].

Subjects were required to meet the following criteria for study entry:

1. Aged 18–75 years and had been diagnosed with asthma for at least 1 year.
2. Pre-bronchodilator forced expiratory volume in 1 s (FEV_1_) of 40–80 % of predicted value at visit 1.
3. Bronchodilator response ≥ 12 % FEV_1_ improvement in response to 400 µg of an inhaled short-acting beta-agonist at visit 1.
4. Using an eligible second controller medication (long-acting beta 2-agonist, long-acting muscarinic antagonist, leukotriene receptor antagonist, or theophylline) within prescribed dosing range for ≥ 6 months prior to visit 1 with no anticipated changes throughout the study.

Subjects who met any of the following criteria prior to enrolment were excluded from the study:

1. Received maintenance oral corticosteroid therapy, defined as daily or alternate day oral corticosteroid maintenance therapy within the preceding 3 months.
2. Treated with systemic (oral, intravenous, or intramuscular) corticosteroids within the preceding 4 weeks.
3. Having a history of cystic fibrosis, chronic obstructive pulmonary disease, or other clinically significant lung disease other than asthma.
4. Being a current smoker or former smoker with a smoking history of > 10 pack-years.

### Intervention

A five-item therapeutic patient education course was created to address learning objectives for asthma control according to the recommendations of the National Standards for Asthma Self-Management Education and the Global Strategy for Asthma Management and Prevention [2, 14]. Patients participated in an initial individual therapeutic patient education course on visit 1 after baseline data collection. The course was provided by a team consisting of an asthma specialist nurse and asthma physician experienced in therapeutic patient education. The therapeutic patient education intervention addressed asthma pathophysiology, illness perceptions, medication skills, self-monitoring techniques, and environmental control and avoidance strategies (reducing exposure). All subjects were required to repeat the therapeutic patient education course in person at the subsequent three visits to the clinic (after 3, 6, and 12 months). Detailed therapeutic patient education course information can be obtained by scanning the WeChat public account link in the supplementary material.

### Atopy

All subjects underwent an epicutaneous prick allergy test for 11 allergen extracts (ALK-Abelló, Hørsholm, Denmark) in the volar surface of the forearm.

### Outcome measures

#### Asthma control

The primary outcome of asthma control was measured by the ACQ-5, Standardized Asthma Quality of Life Questionnaire (AQLQ(S)), and Asthma Symptom Utility Index (ASUI) [15–17]. These short screening instruments have been developed for use in everyday clinical practice, to identify patients with poor asthma control without measuring lung function. For this study, an asthma exacerbation was defined as new or increased asthma symptoms (including wheezing, cough, dyspnea, chest tightness, and nocturnal awakenings because of these symptoms) that led to treatment with systemic corticosteroids or to hospitalization. Treatment with systemic corticosteroids was defined as treatment with oral, intravenous, or intramuscular corticosteroids for at least 3 days or an emergency department visit with at least one intravenous or intramuscular dose of corticosteroids. Medication adherence was calculated as the percentage of prescribed doses taken each week [18].

#### Pulmonary function, fractional exhaled nitric oxide (FeNO) measurement, and serum biomarkers

Subjects completed a pulmonary function test and FeNO measurement at baseline and after 6 and 12 months. Prior to spirometry, airway inflammation was assessed by the FeNO test using a NIOX Flex device (Aerocrine, Solna, Sweden) in accordance with international guidelines [19]. Values are expressed as parts per billion, with a normal FeNO range for healthy adults defined as 5–25 ppb. Spirometry data were obtained by trained respiratory therapists using a portable spirometer (MasterScreen PNEUMO, CareFusion, San Diego, CA, USA) [20]. Serum cytokine levels were assayed using Luminex xMap (Luminex, Austin, TX, USA) and a commercially available cytokine 12-plex panel (Bio-Rad, Hercules, CA, USA) according to the manufacturers’ guidelines and measured on a Bio-Plex 200 platform (Bio-Rad).

### Statistical analysis

We analyzed the data using paired tests comparing asthma control (asthma exacerbation, pulmonary function, and FeNO), quality of life (standardized questionnaire scores), and serum biomarker levels at baseline and 12 months after the initial therapeutic patient education. A paired Student’s *t*-test (or nonparametric Wilcoxon signed-rank test when necessary) was used to compare quantitative variables. Statistical analysis was performed in SPSS version 24 (IBM SPSS, Armonk, NY, USA), and *p* < 0.05 was considered significant.

## Results

### Population

A total of 40 subjects with severe uncontrolled asthma (25 women and 15 men) were included in the study. Mean age was 50.3 years (standard deviation: 11.3). Median duration of asthma diagnosis was 10.5 years, with an interquartile range of 5.0–19.5. Thirty of the 40 patients tested positive for one or several relevant allergens on the skin prick test. Eleven patients had a personal history of atopic dermatitis, 28 suffered from rhinitis, and 16 from conjunctivitis. Table 2 lists patient characteristics at baseline.

**Table 2:** Characteristics of study patients

Characteristics	Total (*n* = 40)
Age (y)	50.3 ± 11.3
Gender (female/male)	25/15
Body mass index (kg/m^2^)	24.1 ± 4.4
Smoking status (never/ex), n	40/0
Atopy, n (%)	30 (75.0 %)
Duration of asthma (y)	10.5 (5.0-19.5)
Combine with rhinitis	28 (70.0 %)
Combine with atopic dermatitis	11 (27.5 %)
Combine with conjunctivitis	16 (40.0 %)
Dose of inhaled corticosteroids (ug/d)	1000(1000–2000)
Budesonide/formoterol, n (%)	24(60 %)
Fluticasone/salmeterol, n (%)	16(40 %)
Montelukast Sodium^a^, n (%)	28(70 %)
Eos in blood(x10^3^/uL)	0.27 ± 0.20

^a^Drugs used in combination. *Eos* eosinophils

### Asthma exacerbation and medication adherence

**Fig. 1:**
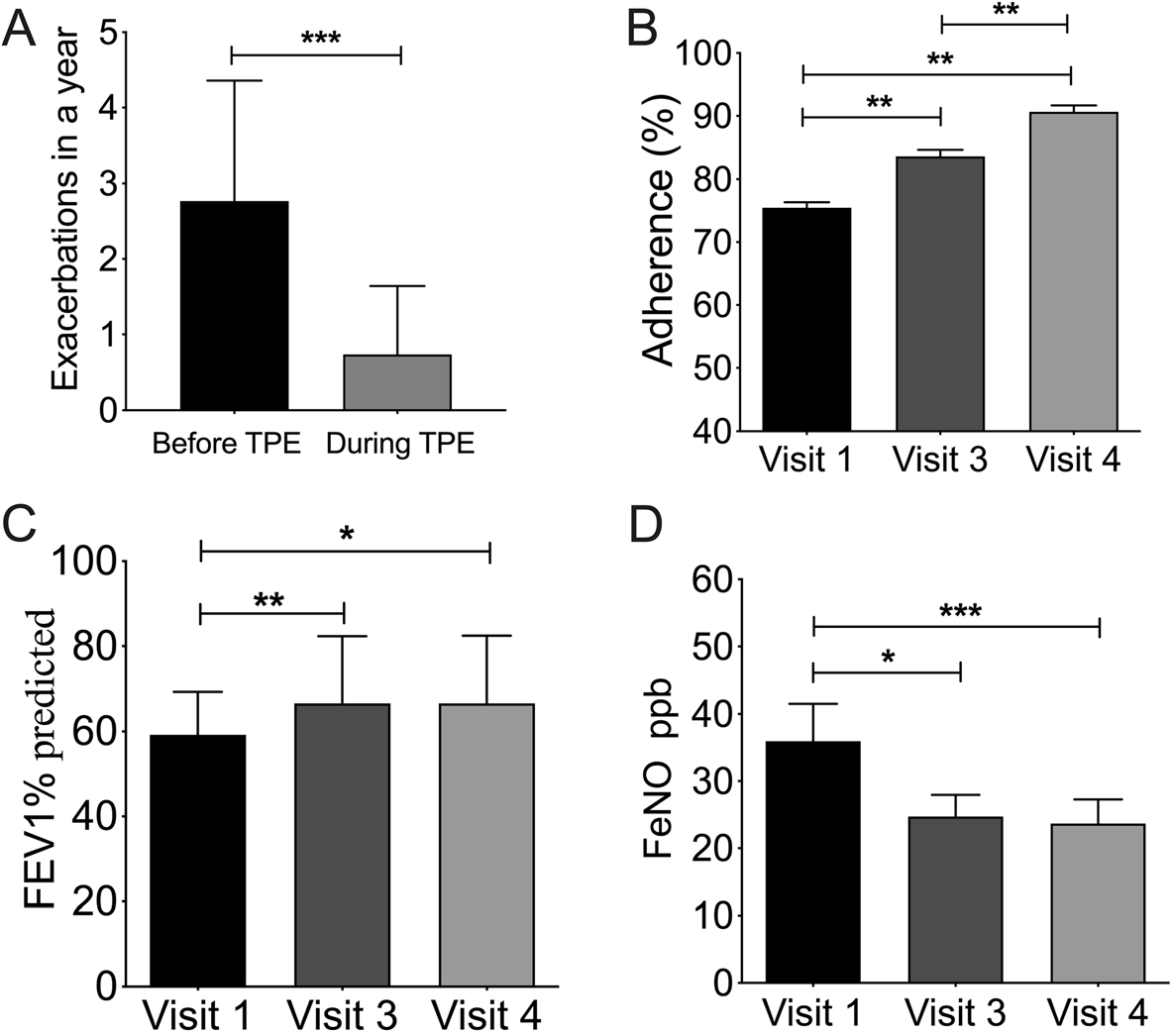
Changes in participant total exacerbations, spirometry, and fractional exhaled nitric oxide (FeNO) over the course of the study. **A** Asthma exacerbations decreased significantly after therapeutic patient education (TPE). **B**–**D** Measurements were conducted on day 1 (visit 1) and after 6 months (visit 3) and 12 months (visit 4). Predicted forced expiratory volume in 1 s (FEV_1_) increased significantly and FeNO decreased over time. Medication adherence was calculated as the percentage of prescribed doses taken each week. *p < 0.05, **p < 0.01, ***p < 0.001

During the 1-year study period, a total of 23 exacerbations had been recorded in 14 patients, seven of whom required emergency treatment and two of whom required hospitalization. The average number of exacerbations was 0.58 per person, and each exacerbation lasted an average of 8.4 days. Asthma exacerbations during the study period decreased significantly from the number of exacerbations reported for the preceding 1-year period (2.80; *p* < 0.001; Fig. 1 A). Mean (± standard deviation) medication adherence for all patients at visits 1, 3, and 4 was 75.5 % ± 6.7 %, 83.6 % ± 7.2 %, and 90.5 % ± 6.0 %, respectively, indicating improved adherence over time (*p* < 0.01; Fig. 1B).

### Pulmonary function and FeNO

Over the course of the study, pulmonary function improved and airway inflammation decreased significantly in the 40 patients. Predicted FEV_1_ increased from 59.2 % ± 1.8 % at baseline to 66.6 % ± 2.9 % after 6 months (*p* = 0.01) and to 66.6 % ± 3.0 % (*p* = 0.011) after 12 months (Fig. 1 C). Similarly, FeNO levels decreased from 36.0 ± 5.6 ppb at baseline to 24.7 ± 3.2 ppb (*p* = 0.023) after 6 months and to 23.7 ± 3.6 ppb (*p* = 0.001) after 12 months (Fig. 1D).

## Quality of life

**Fig. 2:**
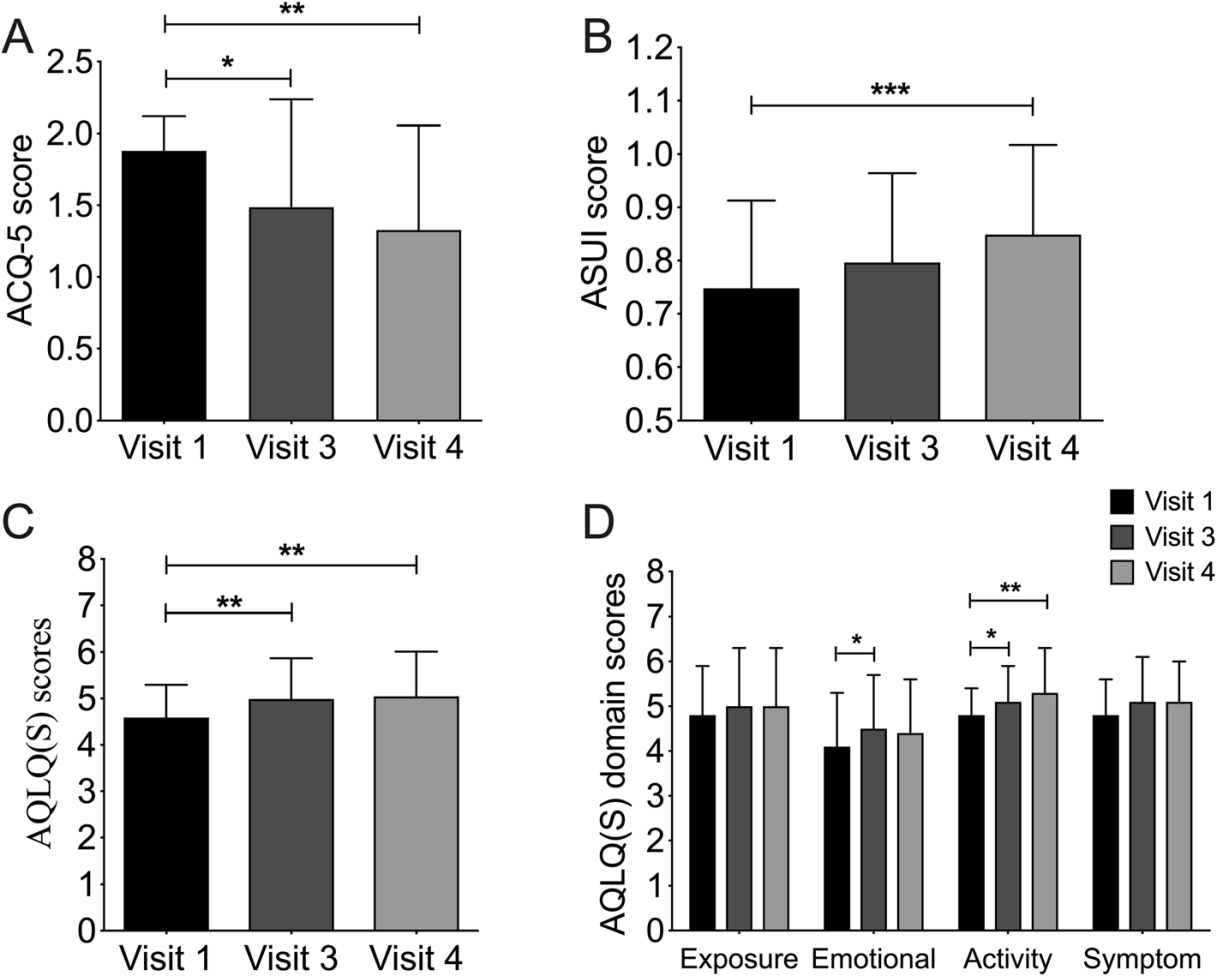
Asthma symptoms, level of asthma control, and asthma-related quality of life. **A** Asthma Control Questionnaire 5 scores decreased significantly from visit 1 (day 1) to visit 3 (6 months after baseline) and visit 4 (12 months after baseline). Asthma Symptom Utility Index scores (**B**) and Standardized Asthma Quality of Life Questionnaire scores (**C**) increased over time. **D** Standardized Asthma Quality of Life Questionnaire scores in four domains increased over time, especially the activity domain score after 6 and 12 months and the emotional domain score after 6 months. *p < 0.05, **p < 0.01, ***p < 0.001

Six months after beginning the study, the questionnaire scores of the 40 patients (ACQ-5, AQLQ(S), and ASUI) indicated an improvement of perceived quality of life. ACQ-5 scores decreased significantly between visit 1 (1.9 ± 0.2) and visit 4 (1.3 ± 0.7; Fig. 2 A). ASUI scores increased from visit 1 (0.75 ± 0.16) to visit 3 (0.80 ± 0.17, *p* = 0.132) and visit 4 (0.85 ± 0.17, *p* = 0.008; Fig. 2B). Total AQLQ(S) scores increased during the study period (*p* < 0.01; Fig. 2 C), and in particular the activity domain scores and emotional domain scores (Fig. 2D).

### Serum biomarkers

**Fig. 3:**
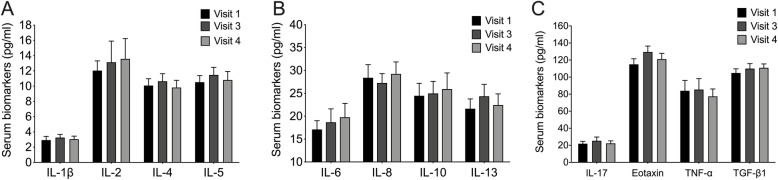
Changes in serum levels of asthma-related biomarkers. There were no significant changes over time between serum levels of interleukins (IL), tumor necrosis factor α (TNF-α), eotaxin, or transforming growth factor β1 (TGF-β1). Visit 1, day 1; visit 3, 6 months after baseline; visit 4, 12 months after baseline

Levels of interleukins, tumor necrosis factor α, eotaxin, and transforming growth factor β1 did not change significantly from baseline levels over the study period (Fig. 3).

## Discussion

Therapeutic patient education is a non-pharmacological intervention that assists patients and their families in learning or maintaining relevant skills for managing life with a chronic disease [12, 13, 21], and has been well-studied in asthma, chronic hand eczema, and chronic heart failure [12, 13, 21]. Previous research supports the hypothesis that therapeutic patient education is essential to improving the knowledge of the asthma patient, self-management of the disease, and the prevention of attacks [13]. However, evidence for the efficacy of therapeutic patient education in severe uncontrolled asthma is lacking. We found that therapeutic patient education was effective in improving asthma control, decreasing exacerbations, and reducing activity limitations in patients with severe uncontrolled asthma. Pulmonary function improved significantly and airway inflammation was reduced in the study cohort, and questionnaire scores indicated improvement in quality of life.

International guidelines highlight that asthma control (defined as prevention of chronic symptoms, minimal use of rescue medication, no exacerbations, no emergency care, and maintenance of normal levels of physical activity) is the major goal in the long-term management of asthma and suggest that the level of control should be monitored by both health care professionals and patients themselves at regular intervals [2, 3]. Despite the many available options for managing uncontrolled asthma, a large proportion of asthma patients still experience uncontrolled disease [22]. Numerous factors contribute to uncontrolled asthma, most commonly are poor adherence, poor inhaler technique, and failure to assess control adequately. A review of 24 survey studies revealed that, in general, adolescents and adults with asthma lack knowledge about the underlying causes of asthma symptoms, such as allergies and triggers; have limited knowledge of treatment options, including correct use of medication; and have low expectations of receiving appropriate therapy or of having a positive encounter with their health care professional [23]. Patients with severe uncontrolled asthma who display poor medication adherence may have an increased risk of experiencing poor asthma outcomes [24], and therapeutic patient education can improve disease control in these patients.

Educational interventions covering important aspects of asthma management are required for adequate self-management [25]. Such interventions for children with asthma and their families have been shown to reduce the number of emergency department visits and hospital admissions [26], and may increase medication adherence and consequently reduce the severity and frequency of symptoms, nocturnal awakenings, and activity limitations [27]. Our results show that therapeutic patient education is effective in improving asthma control, decreasing exacerbations, and reducing activity limitations in patients with severe uncontrolled asthma. Notably, we did not detect significant changes in serum levels of asthma biomarkers over time. The non-invasive asthma control indicators (questionnaires, FEV_1_, and FeNO) were more sensitive and convenient for assessing asthma improvement.

The primary limitations of this study are the small sample and the lack of a control group. Without a control group, it is difficult to assess the direct impact of the therapeutic patient education itself on asthma control, quality of life, and asthma exacerbations, as controller medication in addition to the therapeutic patient education would improve these outcomes.

In summary, our standardized therapeutic patient education program appears to be an effective tool for improving asthma control, decreasing exacerbations, and improve activity limitations in patients with severe uncontrolled asthma, but this approach requires a collaborative effort by patients and physicians.

## Conclusions

Therapeutic patient education can be an effective and collaborative tool for improving asthma control and decreasing exacerbations in patients with severe uncontrolled asthma.

## Supplementary Information

**Additional file 1.**

## Data Availability

Data is available upon reasonable request.

## Abbreviations

ACQ-5: Asthma Control Questionnaire 5
AQLQ(S): Standardized asthma quality of life questionnaire
ASUI: Asthma symptom utility index
FEV1: Forced expiratory volume in 1 s
FeNO: Fractional exhaled nitric oxide
TPE: Therapeutic patient education
