# Supplementary figures and images for: Positive change in asthma control using therapeutic patient education in severe uncontrolled asthma: a one-year prospective study
Source: Asthma Res Pract. 2021 Jul 21;7:10. doi: 10.1186/s40733-021-00076-y (PMC8293484; doi:10.1186/s40733-021-00076-y)

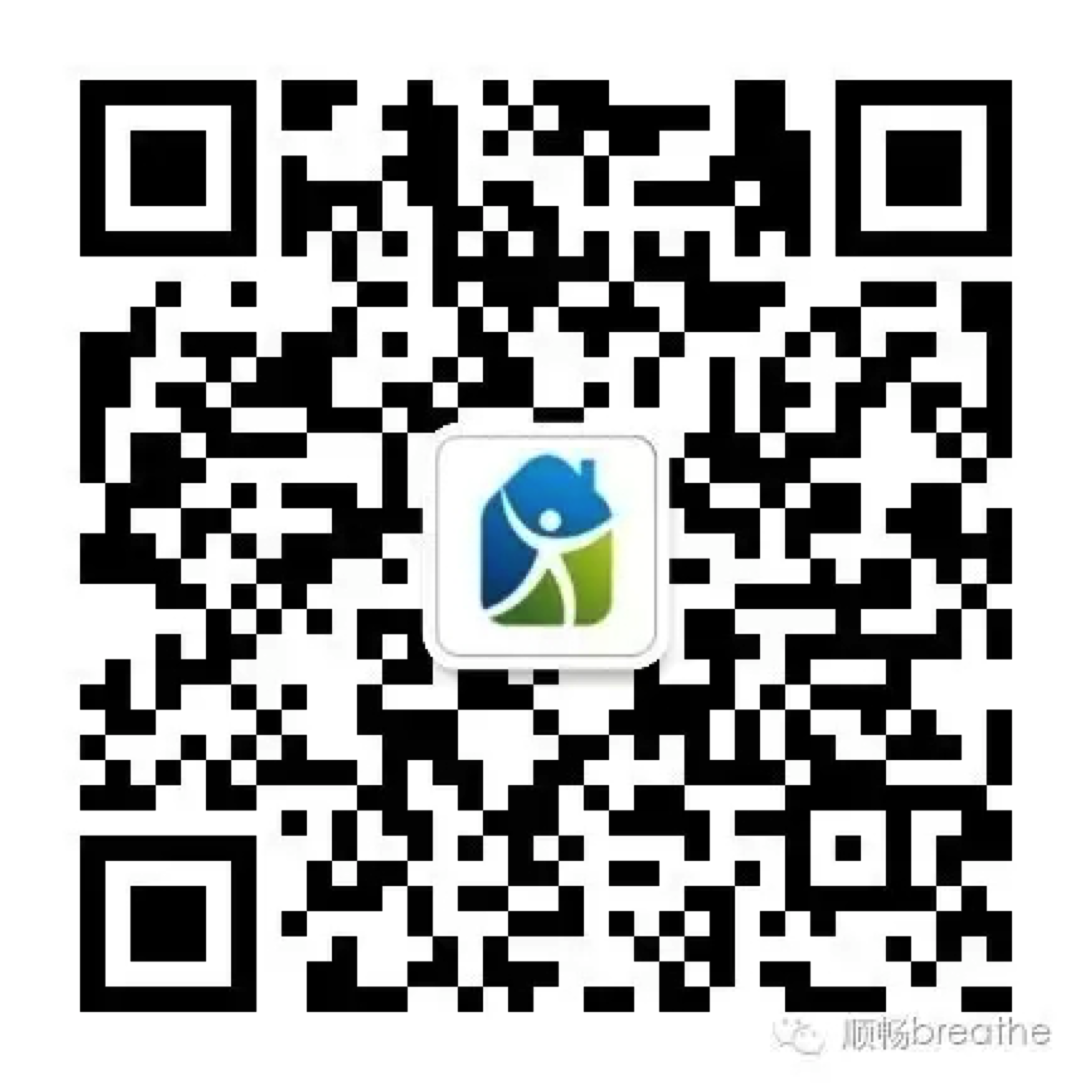

Supplement: Supplementary file 1 — Additional file 1. [file 40733_2021_76_MOESM1_ESM.tiff]
